# Contemporary Treatment Responses of Recurrent Focal Segmental Glomerulosclerosis or Steroid Resistant Nephrotic Syndrome in Children after Kidney Transplantation: Phase 2 of a Multicenter Electronic Health Record Data Analysis

**DOI:** 10.21203/rs.3.rs-9321284/v1

**Published:** 2026-04-20

**Authors:** Vikas R. Dharnidharka, Asha Moudgil, Priya S. Verghese, Leyat Tal, Rebecca R. Scobell, Mahmoud Kallash, Amy J. Goodwin Davies, Nicole Marchesani, Mitchell G. Maltenfort, Megan Kelton, Margret Bock, Eliza Blanchette, Hillarey K. Stone, Caroline Gluck, Frank Hullekes, Leonardo V. Riella, William E. Smoyer, Mark Mitsnefes, Bradley P. Dixon, Joseph T. Flynn, Michael J.G. Somers, Christopher B. Forrest, Susan Furth, Michelle R. Denburg

**Affiliations:** Rutgers Robert Wood Johnson Medical School Department of Pediatrics; Children’s National Medical Center: Children’s National Hospital; Ann and Robert H Lurie Children’s Hospital of Chicago; Texas Children’s Hospital; Childrens Hospital of Philadelphia; Nationwide Children’s Hospital; Childrens Hospital of Philadelphia; Childrens Hospital of Philadelphia; Childrens Hospital of Philadelphia; Seattle Children’s Hospital; Children’s Hospital Colorado; Children’s Hospital Colorado; Cincinnati Children’s Hospital Medical Center; Nemours Children’s Hospital Delaware; Massachusetts General Hospital; Massachusetts General Hospital; Nationwide Children’s Hospital; Cincinnati Children’s Hospital Medical Center; Children’s Hospital Colorado; Seattle Children’s Hospital; Boston Children’s Hospital; Childrens Hospital of Philadelphia; Childrens Hospital of Philadelphia; Childrens Hospital of Philadelphia

**Keywords:** l segmental glomerulosclerosis, kidney transplant, pediatrics, recurrent disease

## Abstract

**Background:**

Recurrence of focal segmental glomerulosclerosis (rFSGS) remains a major complication and a challenge to study treatment efficacy due to lack of granular data in a sufficient sample size. Aggregated data from electronic health records can provide such data.

**Methods:**

We applied computational phenotypes to data from 11 large pediatric health systems in the USA, to identify treatments used and remission outcomes in children with rFSGS after renal transplantation. Additional data were collected by chart review. We performed both linear and non-linear multivariable Cox regression analyses with penalized splines to allow for time-varying predictors. Based on effect sizes from the hazard ratios, we then calculated a sample size needed for a future randomized clinical trial.

**Results:**

Plasmapheresis was used in 101/107 (94%) patients, followed by anti-CD20 agents in 84 (78%), Low-Density-Lipoprotein (LDL)-apheresis in 22 (20%) and CTLA4Igs in 8 (7%). In linear multivariable models, complete remission was associated with more plasmapheresis sessions. In non-linear models, more doses or sessions of all the above treatments were associated with complete remission or any remission (partial or complete). Penalized spline curves for complete or any remission showed greatest yield within 5 doses of anti-CD20 agents but increasing yield with more doses/sessions of CTLA4Igs or LDL-apheresis. Based on observed hazard ratios, a prospective randomized trial of plasmapheresis vs LDL-apheresis would require 155 participants to have 80% power.

**Conclusions:**

Increased doses/sessions or additional therapies for rFSGS associated with more favorable outcomes. Non-linear modelling identified when further increases did not improve outcomes.

## Introduction

Focal segmental glomerulosclerosis (FSGS) is an ultra-rare disease in children, but often has a devastating effect, accounting for 10-15% of pediatric kidney failure [1–3]. Additionally, idiopathic, gene mutation-negative FSGS is characterized by recurrence after kidney transplantation, with reported rates of 40-60% in this specific subset of FSGS [4–6] and no established method of prevention [7–11].

For multiple reasons, managing rFSGS still poses a significant challenge. The rarity of the event has made it very difficult to conduct randomized trials that lead to substantive conclusions. Existing treatment recommendations are therefore based on small study cohorts, case series, and case reports [12, 13]. The heterogeneity of definitions for idiopathic FSGS, disease recurrence, and remission further hinder the interpretation of study results [14]. We do not have any established standard for the treatment of rFSGS post-transplant [15]. Treating physicians are forced to attempt multiple therapies, with wide variability in timing of initiation of treatment, number of sessions if apheresis is used, timing/dosing of depleting antibodies, and concomitant immunosuppressive regimens [14, 16–18]. Many of these therapies are tried together or in varying sequences.

In a prior study, we leveraged data from 7 PEDSnet centers to identify 67 patients with rFSGS and compared them to 98 children with FSGS and kidney transplant in the same time period who did not experience recurrence [19]. In that study we delineated the risk factors for recurrence in a contemporary multicenter cohort, and we found favorable responses to a variety of interventions. PEDSnet has since added more centers, allowing us to conduct an expanded phase 2 study that allowed for further analyses of responses to treatments now reported in larger numbers, and to estimate sample sizes needed for a potetntial future randomized trial.

## Methods

### Study setting and data sources:

We obtained data from centers participating in PEDSnet and/or the Glomerular Learning Network (GLEAN). PEDSnet is a clinical research network that has centralized EHR data from several large pediatric healthcare systems under CHOP IRB protocol #14-011242 [20]. PEDSnet standardizes EHR data across institutions to a common data model, which is derived from the Observational Medical Outcomes Partnership (OMOP) common data model version 5 [21]. GLEAN is a specialty-specific learning network developed in collaboration with PEDSnet that is focused on research and outcomes improvement for children with glomerular disorders (CHOP IRB protocol #16-012878). For this study, we used version 4.0 of the PEDSnet database, which incorporates data from January 2009-December 2022. Data were extracted in December 2020 (phase 1) and December 2022 (phase 2). Phase 1 data came from: Children’s Hospital of Philadelphia; Children’s Hospital Colorado; Cincinnati Children’s Hospital Medical Center; Nationwide Children’s Hospital; Nemours Children’s Health System (a Delaware and Florida health system); Seattle Children’s Hospital; St. Louis Children’s Hospital/Washington University; Phase 2 data added: Children’s Hospital Boston, Children’s National Medical Center, Texas Children’s Hospital and Ann and Robert H. Lurie Children’s Hospital. All participating institutions relied on CHOP’s IRB through the PEDSnet and GLEAN Master Reliance Agreements. Standardized chart review data were collected and managed using Research Electronic Data Capture (REDCap) hosted at Children’s Hospital of Philadelphia [22, 23].

### Cohort Identification:

The initial step in cohort derivation relied on our previously published computable phenotype to identify patients who had at least 3 visits to a nephrology provider and who had nephrotic syndrome [24]. We then refined that search to identify patients with evidence of a kidney transplant between January 1, 2009, to December 31, 2020 (phase 1) or through Dec 31, 2022 (phase 2). We additionally queried locally maintained patient lists to ensure complete capture of all possible cases. We then performed standardized chart review to confirm the diagnosis of idiopathic FSGS. For patients who had multiple kidney transplants within the study period, chart review was restricted to the first transplant. The chart review was also used to confirm treatment for rFSGS and to identify the treatments used, their time course, and kidney allograft outcomes. Medication doses were considered only after the transplant date and prior to allograft loss date, if applicable. Site investigators made the final attributions of complete and partial remission in their chart reviews based on having at least one of the prior published criteria [4, 25, 26].

In phase 1, data extracted from the centralized PEDSnet EHR database included patient demographics and pre-transplant lab values, pre-transplant risk factor covariates, and longitudinal height, weight, serum creatinine, serum albumin, urine protein or albumin, and urine creatinine. All data entries were subject to field validation checks and queries for unexpected values [27, 28]. In phase 2, all data was collected via chart review. All chart reviewers were provided with the same definitions for no remission, partial remission or complete remission as were used in the phase 1 study.

### Statistical Analyses:

Data are presented as frequencies (percentages) for categorical variables, and as means and standard deviations (SD) for continuous variables if following a Gaussian distribution and as medians (inter-quartile range [IQR]) if not. Continuous variables were analyzed by *t*-test or Mann-Whitney U test, and binary and categorical variables by chi-square or Fisher’s exact test, as appropriate. We constructed Kaplan-Meier time to event analyses for remission. Multivariable Cox proportional hazards regression was used to compute the relative hazard for complete remission or any remission (complete or partial; henceforth referred to as complete/partial), with the therapies as time-dependent covariates, modeled with penalized splines (2 degrees of freedom) for number of plasmapheresis sessions, CTLA4-Igs (abatacept or belatacept), anti-CD20 agents, and the number of low-density lipoprotein (LDL) apheresis sessions, to account for fluctuating response rates with increasing doses of a treatment.

The R statistical environment (version 3.5, R Core Team 2022, Vienna, Austria) was used to perform all statistical analyses [29]. Cox modeling was performed using R’s *survival* package [30] and mixed modeling using the *lme4* package [31].

To estimate the size of the treatment effect for a potential prospective study, we adapted the approach described by Hernan et al [32]. Using our Cox models, we plotted predicted average survival functions for two cases: the intact data set where at least one dose of the drug is given (“full drug”) and the same data set with all drug values set to 0 (“no drug”). Based on effect sizes from the hazard ratios, we then calculated a sample size needed for a hypothetical randomized clinical trial from the website https://sample-size.net/sample-size-survival-analysis/.

## Results

We were able to identify 107 children with rFSGS (Table 1). Compared to our phase 1 cohort of 67 patients, the additional 40 children in the phase 2 cohort had a higher rate of native nephrectomy and shorter mean follow-up time. However, all other characteristics were similar between the two subgroups.

Patients received a variety of therapies, including therapeutic plasma exchange (TPE, also known as plasmapheresis); antibodies directed at the CD20 receptor (most commonly rituximab, rarely ofatumumab); LDL apheresis, intravenous immunoglobulin (IVIG), and fusion proteins directed at the CTLA4 receptor (CTLA4-Igs, abatacept or belatacept). These are detailed in Table. Of note, while CTLA4Igs were used in 8 patients, the exact number in Phases 1 and 2 respectively cannot be reported, per PEDSnet policies. However, we are still able to report the p values of associations with remission for CTLA4Igs and the other aforementioned therapies.

Results of our multivariate models stratified by complete remission alone or complete/partial remission are summarized in Table 3. As penalized splines were used to capture potential non-linearities, the p-values were calculated for both the linear component and the non-linear component. In linear models, only TPE was significantly associated with complete remission. In these linear models, none of the therapies were associated with any (complete or partial) remission. However, since these therapies were used in non-random and sequential fashion (with more therapies and higher number of doses more likely if improvement had not occurred), so we must allow that the relationship between treatment and outcome may be to some degree be driven by outcome. As shown in Table 3, all of the therapies were significantly associated with both complete remission alone or complete/partial remission in non-linear models. We have previously reported on time of initiation of each therapy, eGFR decline and allograft loss in the phase 1 study, so those analyses were not repeated for this phase 2 study.

**Figure 1:**
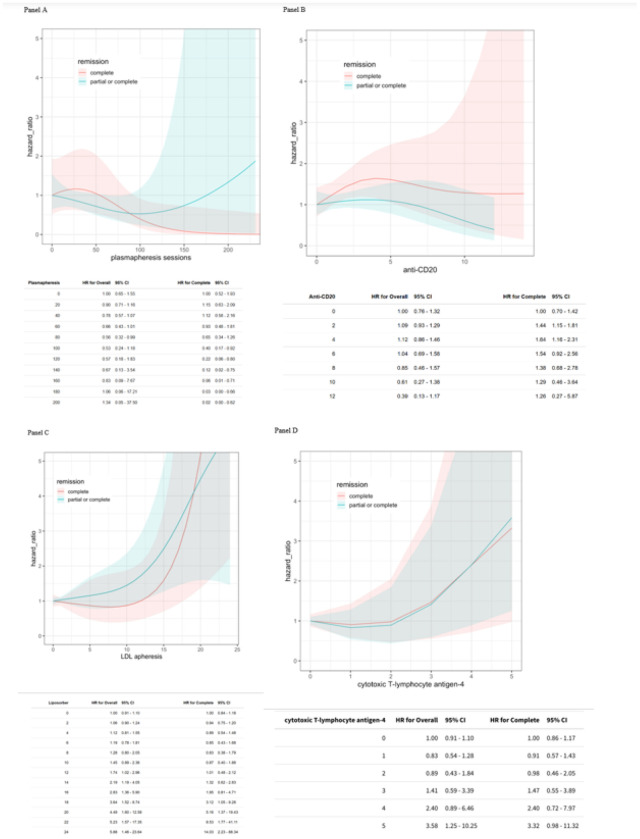


We also explored the responses to individual treatments, which were used in varying non-randomized sequences. Figure 1A shows the spline model with a rising hazard ratio for complete + partial remission after a high number of sessions of TPE. However, the chance of complete remission was above 1 only within the first 50 sessions. For anti-CD20 agents, the hazard ratio for complete remission was greatest within 5 doses, with decreasing benefit thereafter. Notably, the complete remission alone spline curve was much higher than the complete/partial remission spline curve (Figure 1B). The spline curves in Figure 3B show the hazard ratios rise above 1 with greater CTLA4-Ig doses. LDL-apheresis showed hazard ratio curves for both complete remission and complete/partial remission rising above 1 with increasing number of LDL- apheresis sessions (Figure 1C). The same patterns were seen with increasing doses of CTLA4Igs (Figure 1D).

**Figure 2:**
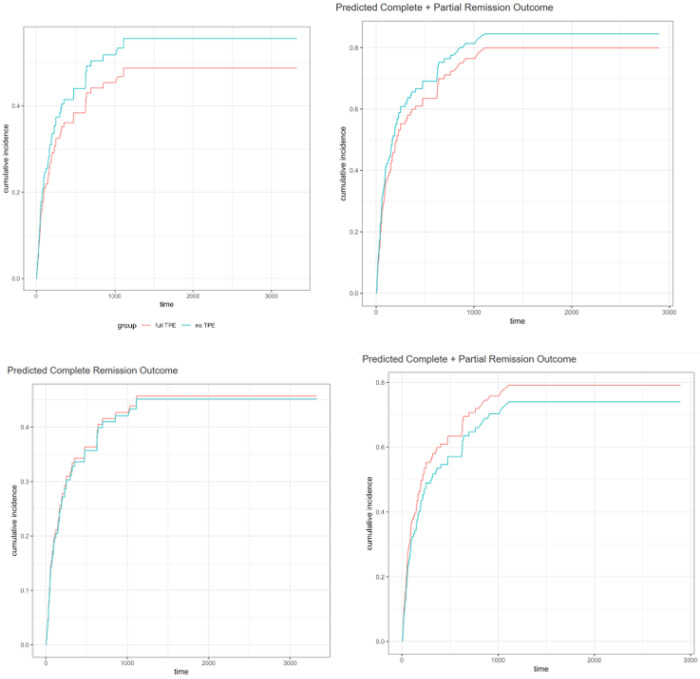
Panel A TPE prediction for complete remission Panel B TPE prediction for complete + partial remission Panel C : LDL-apheresis prediction for complete remission Panel D: LDL-apheresis prediction for complete or partial remission

We then evaluated the timing of remission outcomes in hypothetical comparisons of groups that received TPE versus no TPE, or LDL-apheresis versus no LDL-apheresis. Complete remission was predicted to occur in both TPE or no TPE groups by around 1000 days (Figure 2A). Complete/partial remission was also predicted by around 1000 days in the TPE versus no TPE groups (Figure 2B). Similar predictions were obtained for LDL-apheresis versus no LDL-apheresis for complete remission alone (Figure 2C) or complete/partial remission (Figure 2D).

To estimate an appropriate sample size for a possible randomized controlled trial with 80% power, we used an online calculator (https://sample-size.net/sample-size-survival-analysis/) and parameters based on the current experimental results: a hazard ratio of 1.25 as time to remission for TPE and 5.0 years for LDL-apheresis, resulting in a relative hazard of 4.0 (5.0/1.25) a median time to event of 1.25 years, and a minimum of 17 events of recurrence needed. With these parameters, we estimate a need for 155 subjects (72 in one group and 73 in another) in the 2 groups for 80% power.

## Discussion

This expanded analysis of management of rFSGS at PEDSNet centers confirms our previous findings that favorable outcomes can be achieved using contemporary approaches to this ultra-rare, potentially devastating condition. Additionally, the acquisition of additional patients and data have allowed us to present novel findings of significant association between each intervention and some form of remission in non-linear models. .

A difficulty with rare disease studies is deriving a sufficiently large study cohort that enables robust statistical analysis. The standard error of estimated parameters will decrease with the square root of sample size. So, our phase I study that had 67 patients with rFSGS may not have had sufficient statistical power. The addition of 40 more patients, which would decrease the standard error by about 21% (square root of 67/107 = 0.79), allowed for some findings to change from non-significance to being significantly associated. For instance, in the phase I study, TPE was not associated with complete remission in either linear or non-linear models. Similarly, in phase I the anti-CD20 agents were not associated with complete/partial remission in either linear or non-linear models. In this phase 2 analysis of a larger sample, both agents are now associated with both outcomes of complete remission alone or complete/partial remission in the non-linear models. Gratifyingly, our prior results that showed significant association of LDL-apheresis and CTLA4Igs to both complete remission or complete/partial remission in non-linear models, remained the same in this larger sample, suggesting a robust result. In both phase 1 and phase 2, more associations of significance were found in non-linear models versus the linear models. We believe the non-linear models are more appropriate, given the non-random and varying sequences of therapies applied at varying timepoints, particularly if remission is not achieved with agents used earlier in the course of recurrence.

The spline curves for the association of therapies with remission events also remained similar where the association was previously significant. Thus, the spline curve for TPE for complete/partial remission is very similar in phase 2 to the curve we obtained in phase 1. The depiction of increasing hazard ratio for complete remission alone or complete/partial remission with more sessions of LDL-apheresis or more doses of CTLA4Igs also remained the same in phase 2 analyses. The spline curve for complete remission alone with anti-CD20 agents changed slightly to now have the hazard ratio stay above 1 at all doses, though the same drop in hazard ratio was seen beyond 5 doses.

Novel to this phase 2 study, we attempted to use our aggregated data to predict what the responses would be if a patient with rFSGS received at least one session of TPE or LDL-apheresis, versus either no TPE or no LDL-apheresis, respectively. The curves in Figures 2 suggest that the differences in groups are small and the curves separate out only after an extensive follow up period. Note though that these constructs are highly artificial, since very few patients did not actually receive any TPE, while LDL-apheresis use is still not easy to access. We did not attempt these comparisons with CTLA4Igs due to the small and unreportable sample size, nor with anti-CD20 agents due to the drops in hazard ratios for both curves after 5 doses.

The strengths of our study included the high level of granular detail that could be extracted from the EHR across a multicenter population of an ultra-rare disease. Our data are extracted from a contemporary era and a large representative source population, allowing for greater generalizability than prior smaller studies. Limitations of our study include its retrospective nature, and the individualized treatments used at each center, often in greater combinations of agents for those who did not respond early. Further, the evaluation of treatment response is limited by the extensive use of TPE in almost all patients and by the small number of cases treated with CTLA4 agents. We have adjusted for these clinical variations in assessing responses to agents. We did not collect data on the adverse effects of the therapies used. Furthermore, in our regression analyses, it is assumed that the outcome follows the treatment choice and not vice versa. But in these patients the number of administrations and combinations can increase until the patient responds, so the treatment is a consequence of whether and how early the patient responds. Our application of time-varying predictor methods for censored time-to-event data addresses this to some extent. We also note the limitations associated with the secondary use of EHR data for research, such as non-random missingness and data quality issues [33].

In summary, using real-world multi-center data, we identified a contemporary high-risk cohort of children with rFSGS after kidney transplant and present novel data on treatment responses. All of the major contemporary therapies used were now associated with complete or complete/partial remission, suggesting a more favorable outlook for rFSGS than previously believed. Our data can inform the design and sample size for a future prospective randomized clinical trial for rFSGS between TPE and LDL-apheresis.

## Figures and Tables

**Table 1.** Demographic and clinical characteristics of the recurrent FSGS cohorts

FSGS and kidney transplant	Phase 1	Phase 2	Overall
N (%)	67	40	107
Mean age at transplant in years (SD)	12.4 (4.4)	12.4 (5.0)	12.4 (4.6)
Female Sex %	46.3	32.5	41.1
Ancestry			
African ancestry %	20.9	30.0	24.3
Non-African %	79.1	70.0	75.7
> 1 atopy diagnosis (%)	25.4	25.0	25.2
Donor source living related %	26.9	22.5	25.2
Pre-transplant native nephrectomy Yes %	16.4	57.5	31.8
Mean follow up in years (SD)	7.4 (3.7)	4.6 (3.5)	6.4 (3.9)
HLA DR7 allele presence in recipient %	26.9	37.5	30.8

**Table 2.** Treatments received for recurrent FSGS. These groups are not mutually exclusive, and many patients received 3 or more therapies.

Treatment type	Phase 1 (n = 67)	Phase 2 (n = 40)	Overall (n = 107)
TPE	65 (97)	36 (90)	101 (94)
Anti-CD20 agents	56 (84)	28 (70)	84 (79)
TPE + anti-CD20 agent	54 (81)	26 (65)	80 (75)
Low density lipoprotein apheresis	-	-	22 (21)
CTLA4Igs	-	-	8 (7)

TPE: therapeutic plasma exchange. Figures in parentheses represent percentages.

CTLA4Igs were used in < 11 patients in both phase 1 and 2 and overall. TPE without these other therapies was used in 20 patients. TPE + anti-CD20 agent + low density lipoprotein apheresis was used in 16 patients. All other combinations were used in < 5 patients.

**Table 3.** P-values for significance from the linear and non-linear multivariate models for the different treatments used, by complete + partial or complete remission. Separate p-values are given for linear and non-linear components. All agents were significantly associated in non-linear models with either complete remission alone or complete + partial remission. In linear models, only TPE was associated with complete remission.

	Complete + Partial Remission		Complete Remission	
	P value (linear)	P value (non-linear)	P value (linear)	P value (non-linear)

TPE	0.162	**0.025**	**<0.001**	**0.002**
CTLA4-lgs	0.221	**<0.001**	0.258	**0.025**
LDL-apheresis sessions	0.116	**0.005**	0.296	**<0.001**
Anti-CD20 agent	0.164	**0.018**	0.923	**0.004**

TPE = therapeutic plasma exchange, also known as plasmapheresis; CLTA4-Igs = abatacept or belatacept, LDL = low density lipoprotein.

## Data Availability

The EHR data used for this study were derived from a HIPAA limited dataset among patients receiving care at a participating institution. Consent for this study was waived. As a result, the patient-level data cannot be shared with other investigators. However, the authors are willing to share the R code used to generate these results.

## Abbreviations

ANZDATA: Australia and New Zealand Dialysis and Transplant Registry
eGFR: estimated glomerular filtration rate
EHR: electronic health record
FSGS: focal segmental glomerulosclerosis
HR: hazard ratio
IQR: interquartile range
KTx: kidney transplant
LDL-A: low density lipoprotein apheresis
NAPRTCS: North American Pediatric Renal Trials and Collaborative Studies
rFSGS: recurrent focal segmental glomerulosclerosis
SD: standard deviation
TANGO: post TrANsplant GlOmerular Consortium
TPE: therapeutic plasma exchange, also known as plasmapheresis
USRDS: United States Renal Data System
